# Effect of response format for clinical vignettes on reporting quality of physician practice

**DOI:** 10.1186/1472-6963-9-128

**Published:** 2009-07-28

**Authors:** Thao Pham, Carine Roy, Xavier Mariette, Fréderic Lioté, Pierre Durieux, Philippe Ravaud

**Affiliations:** 1 Department of Rheumatology, CHU Conception, 13005 Marseille, France; 2 Department of Epidemiology, biostatistics and clinical research, CHU Bichat, 13018 Paris, France; 3 Department of Rheumatology, CHU Bicêtre, Le Kremlin-Bicêtre, France; 4 Department of Rheumatology, CHU Lariboisière, 75 Paris, France; 5 Department of public health and medical informatics, Hôpital Européen Georges Pompidou and Paris Descartes University, Paris, France

## Abstract

**Background:**

Clinical vignettes have been used widely to compare quality of clinical care and to assess variation in practice, but the effect of different response formats has not been extensively evaluated. Our objective was to compare three clinical vignette-based survey response formats – open-ended questionnaire (A), closed-ended (multiple-choice) questionnaire with deceptive response items mixed with correct items (B), and closed-ended questionnaire with only correct items (C) – in rheumatologists' pre-treatment assessment for tumor-necrosis-factor (TNF) blocker therapy.

**Methods:**

***Study design***: Prospective randomized study. ***Setting***: Rheumatologists attending the 2004 French Society of Rheumatology meeting. Physicians were given a vignette describing the history of a fictitious woman with active rheumatoid arthritis, who was a candidate for therapy with TNF blocking agents, and then were randomized to receive questionnaire A, B, or C, each containing the same four questions but with different response formats, that asked about their pretreatment assessment. ***Measurements***: Long (recommended items) and short (mandatory items) checklists were developed for pretreatment assessment for TNF-blocker therapy, and scores were expressed on the basis of responses to questionnaires A, B, and C as the percentage of respondents correctly choosing explicit items on these checklists. ***Statistical analysis***: Comparison of the selected items using pairwise Chi-square tests with Bonferonni correction for variables with statistically significant differences.

**Results:**

Data for all surveys distributed (114 As, 118 Bs, and 118 Cs) were complete and available for analysis. The percentage of questionnaire A, B, and C respondents for whom data was correctly complete for the short checklist was 50.4%, 84.0% and 95.0%, respectively, and was 0%, 5.0% and 5.9%, respectively, for the long version. As an example, 65.8%, 85.7% and 95.8% of the respondents of A, B, and C questionnaires, respectively, correctly identified the need for tuberculin skin test (p < 0.0001).

**Conclusion:**

In evaluating clinical practice with use of a clinical vignette, a multiple-choice format rather than an open-ended format overestimates physician performance. The insertion of deceptive response items mixed with correct items in closed-ended (multiple-choice) questionnaire failed to avoid this overestimation.

## Background

Improvement of quality of clinical practice needs quality measurements. These measurements must be accurate, valid and feasible. The advantages and disadvantages of different methods of measuring the process of care, including both the competence of the clinician and what the clinician actually does, have been well described. Methods include chart extraction, standardized patients and clinical vignettes. Substantial inaccuracies in administrative data are common, which leads to expensive data extraction and difficulty in validating data [1-3]. Compared to standardized patients and chart extraction, clinical vignettes are an accurate, valid, feasible and inexpensive tool to measure quality of health care [4,5]. Thus, clinical vignettes have been used widely to compare quality of clinical care and to assess variation in practice across countries, health care systems, specialties or clinicians [6-11].

Vignette-based surveys for physicians feature open-ended questions rather than multiple-choice questions or checklists. In this way, physicians can give a personal response to each question, which ensures that the survey captures the full range of practice variation [11,12]. Open-ended questions avoid the "cueing" inherent in multiple-choice questions, which could overestimate real physician performance. However, the accuracy of close-ended questionnaires has been demonstrated in several domains: the format provides a significantly higher rate of accuracy than an open-ended format in terms of eyewitness confidence with the former format [13]. Close-ended questionnaires maximize questionnaire response rate and ensure questionnaire completeness [14].

Moreover, different formats yield different answers: in one study, a closed-ended questionnaire produced results reflecting higher willingness to pay for a health care intervention, with different justifications for those evaluations than did those from an open-ended one [15]. A public opinion survey with two different response modalities asked subjects about the most important problem facing the United States: respondents of the open-ended format most often complained about political leadership, whereas those of the close-ended format considered violence as most important [16].

From these data, we wanted to evaluate how clinical vignette-based surveys influence physician responses. Assuming that closed-ended (multiple-choice) questions for vignettes produce different responses than open-ended, leading to an overestimation of professional performance, we aimed to determine whether the influence of deceptive response items included in the closed-ended questionnaires result in a better assessment of professional performance.

## Methods

We conducted a prospective randomized study aimed at comparing three response modalities for a vignette-based survey: open-ended questionnaire, closed-ended questionnaire (with only correct response items) and closed-ended questionnaire with deceptive response items mixed with correct items.

### Survey

The survey was composed of two parts. The first part was short, identical in each questionnaire, and collected demographic characteristics and specialties of physicians. The second part was the clinical vignette.

### Vignette

The vignette reported the history of a fictitious 50-year-old woman with active rheumatoid arthritis, a candidate for therapy with tumor necrosis factor (TNF) blocking agents. Physicians were asked to answer four questions about their pre-treatment assessment, considering that TNF-blocking treatment was planned: 1) what specific data are you searching for in this patient's history? 2) What clinical data are you personally searching for during the physical examination? 3) Which biological, radiographic or other tests do you request? 4) What other preventive measures do you take? Physicians were given these questions in one of three questionnaire formats: open-ended questionnaire (questionnaire A) [see Additional file 1], closed-ended (multiple-choice) questionnaire with deceptive response items mixed with correct items (n = 73) (questionnaire B) [see Additional file 2], closed-ended questionnaire with only correct items (n = 35) (questionnaire C) [see Additional file 3]. Deceptive and correct response items were created by following published international and national recommendations to help physicians care for patients under this treatment [17-21]. Three experts (XM, TP and FL) met to formulate correct and deceptive items. They based their work on the published international and national recommendations to help physicians care for patients under this treatment, to first determine the correct items, and then propose deceptive items. Each expert has elaborated 20 deceptive items, within 4 categories: patient's history, physical examination, biological, radiographic or other tests and other preventive measures. From the 53 elaborated items (duplicates were eliminated), only the more believable were kept, allowing to propose 38 deceptive items, which were mixed with the 35 correct items in questionnaire B.

### Scoring

Responses to questionnaire A were coded for comparison to those of the other two questionnaires. For each item, the response was classified as "item correctly selected"; "item incorrectly selected"; "item correctly not selected"; "item incorrectly not selected." We classified each item according to three sources: an evidence-based literature search of clinical practice concerning TNF-blocking drug management, international and national guidelines, and a French clinical tool guide on use of TNF-blocking agents elaborated by an expert panel of academic and community physicians [22]. From these sources, we developed two checklists of items for pretreatment assessment for TNF-blocker therapy: a long version extracted from the French clinical tool guide on use of TNF-blocking agents [22], with detailed data on research into possible contraindications (Table 1), and a short version extracted from the same clinical tool guide and from the French agency for health care recommendations with items mandatory in France (Table 2) [23].

**Table 1:** Long version of the checklist of correct items in pretreatment assessment for TNF-blocker therapy extracted from the French clinical tool guide [22]:

Question 1: What specific data are you searching for in this patient's history?
History of primary tuberculosis infection or of visceral tuberculosis

History of vaccination with BCG (bacille Calmette-Guerin)

History of positive response to intradermal tuberculin testing

History of correct treatment for possible active tuberculosis

History of infection: cutaneous

History of infection: pulmonary

History of infection: urinary

History of infection: septicemia

History of infection: septic arthritis

History of neurologic disease

History of neoplasia or of hemopathy

Question 2: What clinical data are you personally searching for during the physical examination?

Lymphadenopathy

Neurologic abnormalities

Question 3: Which biological, radiolographic or other tests do you request?

Blood count

Serum protein electrophoresis

Liver function tests

Hepatitis C serology

HIV serology

Antinuclear antibodies

Chest x-ray

Question 4: What other preventive measures do you take?

Tuberculin skin test

**Table 2:** Short version of the checklist of correct items in pretreatment assessment for TNF-blocker therapy extracted from the French agency for health care recommendations (mandatory in France)[23]:

Question 1: What specific data are you searching for in this patient's history?
History of tuberculosis

Question 2: What clinical data are you personally searching for during the physical examination?

None

Question 3: Which biological, radiographic or other tests do you request?

Chest x-ray

Question 4: What other preventive measures do you take?

Tuberculin skin test

### Questionnaire administration

During the 2004 French Society of Rheumatology meeting, rheumatologists were asked to participate in a survey concerning pretreatment assessment in cases of therapy with TNF blockers, which aimed at detecting contraindications to treatment. The survey was conducted on behalf of the *Club Rhumatismes et Inflammation *(CRI), the division of the French Society of Rheumatology dedicated to musculosquelettal inflammatory diseases.

Until the targeted sample size was achieved, the survey distribution was randomized, with each physician receiving only one questionnaire format (A, B or C). Rheumatologists were blinded to the hypothesis. Particularly, they were unaware of the existence of different response modalities, of deceptive items in questionnaire B, and that all items of questionnaire C were correct. Time to complete the survey was limited to fifteen minutes.

Four interviewers were responsible for encouraging participation in the survey, explaining the official nature of the survey, checking that all the questionnaires were correctly completed in the time allowed, and checking the randomization was achieved. Participation was voluntary, and physicians' responses were kept anonymous.

### Statistical analysis

A chi-square test was used to compare the proportion of items selected or not in terms of the questionnaire format of each of the three questionnaires. A p < 0.05 was considered statistically significant. Pairwise chi-square tests with Bonferonni correction (corrected significant probability of 0.017) were used to compare variables with statistically significant differences. Statistical analysis involved use of SAS Release 8.2 and Splus 6.2.

Sample size calculation: Three sets of 100 questionnaires – one set for each of the three questionnaires – were planned for the analysis. In fact, when considering pairwise comparisons for the response item "tuberculin skin test," with a sample size of 100 in two groups, a two-group chi-square test with a 0.017 two-sided significance level would have 80% power to detect a difference between a 65% proportion in one group and a 85% proportion in the other group. Because we expected 15% incomplete or non-analyzable questionnaires, we distributed 350 questionnaires.

## Results

### Respondents

Of 350 questionnaires dispensed (114 questionnaire As, 118 questionnaire Bs and 118 questionnaire Cs), all were completed, and all responses were eligible for further analysis. Table 3 displays demographic and specialty characteristics of physicians responding to the identical format part of the survey. Physicians were similar in terms of sex, practice duration and practice modalities. Questionnaire A respondents were younger than those of the other two questionnaires. Only two questions were asked about rheumatologists' experience with TNF-blocking drugs: 69.4% had already prescribed anti-TNF therapy and 43.1% had access to a checklist for screening potential contraindications in their department.

**Table 3:** Demographic characteristics and specialties of physicians completing questionnaires A, B and C.

Physician characteristics		Questionnaire A (open-ended)	Questionnaire B (closed-ended with deceptive items)	Questionnaire C (closed-ended)
N		114	118	118

Sex (% female)		41.2	37.3	37.3

Age (mean ± SD)		43.8 ± 9.2	46.4 ± 8.8	47.2 ± 9.6

Years practicing rheumatology (mean ± SD)		17.8 ± 8.6	18.7 ± 8.5	19.4 ± 8.9

	Exclusive private practice (%)	35.0	32.8	31.1

Practice	Both private and public practice (%)	26.5	32.8	28.6

	Exclusive public practice (%)	38.5	34.4	40.3

Previously prescribed TNF-blocking drug (yes, %)		64.9	68.6	73.9

Access to a checklist before TNF-blocker drug prescription (yes, %)		46.6	44.5	51.9

Questionnaire A: open-ended; Questionnaire B: closed-ended with deceptive items; Questionnaire C: closed-ended

### Questionnaire responses

Although we expected 15% incomplete or non-analyzable questionnaires, we did not observe any missing data for open-ended or closed-ended questionnaires.

Significant differences depending on questionnaire format were found in reporting pre-treatment assessment. Compared with the two closed-ended questionnaires, the open-ended questionnaire gave lower reporting of items correctly selected and correctly not selected (Table 4).

**Table 4:** Percentage of physicians who correctly selected all the items of the short or long checklist for pretreatment assessment for TNF-blocker therapy and to specific items, by questionnaire A, B and C and pairwise chi-square comparison

	Questionnaire A	Questionnaire B	Questionnaire C	A and B comparison p-value	A and C comparison p-value	B and C comparison p-value	A, B and C comparison p-value
Long version of checklist	0.0	5.0	5.9	0.0293	0.0143	1.0000	0.0167

Short version of checklist	50.4	84.0	95.0	<0.0001	<0.0001	<0.0001	0.0060

Chose item "chest X-rays"	84.6	96.6	98.3	0.0015	0.0002	0.6835	<0.0001

Chose item "tuberculin skin test"	65.8	85.7	95.8	0.0004	< 0.0001	0.0072	<0.0001

Questionnaire A: open-ended; Questionnaire B: closed-ended with deceptive items; Questionnaire C: closed-ended

In terms of global results, none of the questionnaire A respondents proposed response items of the long checklist, although 5.0% and 5.9% of questionnaire B and C respondents, respectively, correctly selected all items (Table 4).

For the A, B and C questionnaires, 50.4%, 84.0% and 95.0% respondents, respectively, correctly selected all mandatory response items of the short checklist. When focusing on response items within the short checklist, questionnaires B and C did not produce differences in responses to item "order chest X-rays" also difference was observed with open- and close-ended questionnaires (p < 0.0001) (Table 4). In contrast, respondents to questionnaires A, B, and C significantly differed in responses for another mandatory item, "obtaining a tuberculin skin test": 65.8%, 85.7% and 95.8% respondents, respectively, identified this item.

Rheumatologists completing the closed-ended questionnaire B, with deceptive response items, more often chose these items, such as seeking advice of a systematic lung specialist (26.1%) or determining blood sugar level (40.3%). None of the questionnaire A respondents spontaneously proposed these items. Questionnaire B respondents showed a tendency for a lower percentage of correctly selected items than questionnaire C respondents. The open-ended format allowed for collecting qualitative data on items that we did not propose in the close-ended questionnaires, such as "give information to the patient on potential adverse effects" or "give information to the patient on monitoring these drugs."

## Discussion

We compared three clinical vignette-based survey response formats: an open-ended questionnaire, a closed-ended (multiple-choice) questionnaire with cued correct items and a closed-ended questionnaire with deceptive items mixed with correct items. As expected, use of a closed-ended questionnaire with cued items overestimated physicians' performance as compared with an open-ended questionnaire, given that the latter is considered as the gold standard in assessing practice [5,12,24]. Also as expected, the open-ended questionnaire supplied more information on clinical practice than the close-ended questionnaires; physicians were more willing to provide information to the patient. Although we included response items on examinations or tests, such as cutaneous examination, in the closed-ended questionnaires, none of the respondents of the open-ended questionnaire suggested such tests.

Our study focuses on the difficulty in evaluating the quality of physician performance for specific domains with open-ended questionnaires. Physicians may be more brief with open-ended formats and responses may be less accurate. Of the 114 questionnaire A respondents, 74.4% responded with "tuberculosis" to the "other tests" question but gave no specific description of a test or what clinical examination they would do to evaluate this tuberculosis risk. In the closed-ended format, we assumed that including deceptive items would influence respondents' answers and lower the overestimation inherent in the closed-format survey. To our knowledge, this is the first time that deceptive items have been mixed with cued items in a close-ended questionnaire. Questionnaire B respondents indeed selected fewer correct items than did questionnaire C respondents. However, these results were very different from those obtained with the open-ended questionnaire (A), which are probably closer to reality.

The influence of framing questionnaire items remains crucial for clinical practice evaluation. This bias in response acquiescence has been reported from study of two versions of a training satisfaction questionnaire randomly distributed to medical residents; in one, half the items were stated positively and half negatively, and in the other, all items were stated positively. Results showed a significant effect of positive versus negative framing [25].

## Conclusion

In conclusion, even if closed-ended questionnaires may provide more accurate data in clinical practice evaluation, general open-question format has value in such evaluation. Strategies for generating quantitative and qualitative data from open-ended questionnaires, associated or not with closed-ended questionnaires, facilitating survey analysis, are very likely interesting to develop to improve physician performance evaluation [25].

## Competing interests

This study was support by an educational grant from Wyeth Pharmaceuticals. The pharmaceutical company has paid for the 3-day employment of the 4 Ipsos interviewers. Ipsos is an independent company focused on survey-based research. The authors did not receive money from the pharmaceutical company.

## Authors' contributions

TP participated in the design of the study, carried out the survey, participated in the elaboration of the clinical vignettes and drafted the manuscript. CS performed the statistical analysis. XM and FL participated in the elaboration of the clinical vignettes and especially the formulation of the correct and deceptive items. PD participated in the design of the study and its coordination. PR conceived of the study, and participated in its design and coordination. All authors read and approved the final manuscript.

## Pre-publication history

The pre-publication history for this paper can be accessed here:

http://www.biomedcentral.com/1472-6963/9/128/prepub

## Supplementary Material

Additional file 1 **Questionnaire A**. Vignette reporting history of a fictitious 50-year-old woman with active rheumatoid arthritis, candidate for therapy with tumor necrosis factor (TNF) blocking agents, with an open-ended questionnaire about pre-treatment assessment. Click here for file

Additional file 2 **Questionnaire B**. Vignette reporting history of a fictitious 50-year-old woman with active rheumatoid arthritis, candidate for therapy with tumor necrosis factor (TNF) blocking agents, with a closed-ended (multiple-choice) questionnaire about pre-treatment assessment containing deceptive response items mixed with correct items. Click here for file

Additional file 3 **Questionnaire C**. Vignette reporting history of a fictitious 50-year-old woman with active rheumatoid arthritis, candidate for therapy with tumor necrosis factor (TNF) blocking agents, with a closed-ended (multiple-choice) questionnaire about pre-treatment assessment containing only correct items. Click here for file
