# Supplementary material for: Effect of response format for clinical vignettes on reporting quality of physician practice
Source: BMC Health Serv Res. 2009 Jul 28;9:128. doi: 10.1186/1472-6963-9-128 (PMC3224732; doi:10.1186/1472-6963-9-128)
Supplement: Additional file 1 — Questionnaire A. Vignette reporting history of a fictitious 50-year-old woman with active rheumatoid arthritis, candidate for therapy with tumor necrosis factor (TNF) blocking agents, with an open-ended questionnaire about pre-treatment assessment. [file 1472-6963-9-128-S1.pdf]

Club Rhumatismes & Inflammations

Numéro d'anonymat : A

Enquête du CRI

Traitements par agents anti-TNF : quel bilan pré-thérapeutique ?

Frédéric Lioté, Thao Pham, Philippe Ravaud

Les traitements anti-TNF sont prescrits chez des patients atteints de PR ou de spondylarthopathies. Les indications sont actuellement libellées selon les AMM. Ils exposent à un certain nombre d'effets secondaires de sévérité variable pour lesquels il n'y a actuellement en France que peu de recommandations de pratiques ou de prescription d'examens pré-thérapeutiques. Chaque clinicien évalue le cas particulier de son patient selon sa propre expérience.

Au nom du CRI, nous vous proposons de répondre à une enquête anonyme à partir d'un cas clinique, simple et rapide, sur vos pratiques cliniques et les mesures que vous prenez chez vos patients avant prescription d'un traitement anti-TNF. Il s'agit de faire un instantané, un état des lieux des pratiques réelles dans notre pays.

Age : |__|__| ans

Sexe F ☐ M ☐

Année d'obtention de la spécialité : |__|__|__|__|

Membre de la SFR : Oui ☐ Non ☐

Modalités actuelles d'exercice :

- libéral exclusif ☐
- praticien attaché hospitalier et exercice libéral ☐
- hospitalier temps plein ☐ temps partiel ☐
- hospitalo-universitaire (PU-PH, PHU, MCU-PH) ☐ CCA ☐ interne de spécialité ☐

Avez-vous déjà prescrit vous-même un traitement anti-TNF ?

Oui ☐ Non ☐

Dans le service hospitalier où vous exercez en consultation ou en salle, disposez-vous d'une liste systématique d'items et d'examens à vérifier avant prescription d'un anti-TNF ?

Oui ☐ Non ☐ Ne sait pas ☐

Numéro d'anonymat : A

Femme, 50 ans, polyarthrite rhumatoïde séropositive (FR IgM) depuis 7 ans avec :

- Echappement à une bithérapie MTX (20 mg/sem PO) + Salazopyrine (2 g/j) + corticothérapie de 10 mg/j + AINS conventionnel à pleine dose.
- Antécédents :
  - ménopause depuis 1 an non substituée, fibro-adénome mammaire
  - hypothyroïdie secondaire à une maladie de Hashimoto
  - Fille de 29 ans atteinte de la même dysthyroïdie
  - Fracture de côte après chute au décours d'un vertige de Ménière
- Examen clinique
  - Pression artérielle = 120/76, auscultation cardiopulmonaire normale
  - Absence de nodule rhumatoïde ou de signe de vascularite

Vous voulez traiter cette patiente par un agent anti-TNF. Quel est votre bilan pré-thérapeutique (en dehors du bilan d'activité ou de sévérité de la maladie) ?

1. Quelles données spécifiques recherchez-vous dans les antécédents de votre patiente ?

2. Quelles données de l'examen physique recueillez-vous ?

Numéro d'anonymat : A

3. Quels examens biologiques, radiologiques et autres prescrivez-vous ?

4. Quelles autres mesures préventives prenez-vous ?
