# Supplementary material for: Effect of response format for clinical vignettes on reporting quality of physician practice
Source: BMC Health Serv Res. 2009 Jul 28;9:128. doi: 10.1186/1472-6963-9-128 (PMC3224732; doi:10.1186/1472-6963-9-128)
Supplement: Additional file 2 — Questionnaire B. Vignette reporting history of a fictitious 50-year-old woman with active rheumatoid arthritis, candidate for therapy with tumor necrosis factor (TNF) blocking agents, with a closed-ended (multiple-choice) questionnaire about pre-treatment assessment containing deceptive response items mixed with correct items. [file 1472-6963-9-128-S2.pdf]

Club Rhumatismes & Inflammations

CRI

Numéro d'anonymat : B

Enquête du CRI

Traitements par agents anti-TNF : quel bilan pré-thérapeutique ?

Frédéric Lioté, Thao Pham, Philippe Ravaud

Les traitements anti-TNF sont prescrits chez des patients atteints de PR ou de spondylarthopathies. Les indications sont actuellement libellées selon les AMM. Ils exposent à un certain nombre d'effets secondaires de sévérité variable pour lesquels il n'y a actuellement en France que peu de recommandations de pratiques ou de prescription d'examens pré-thérapeutiques. Chaque clinicien évalue le cas particulier de son patient selon sa propre expérience.

Au nom du CRI, nous vous proposons de répondre à une enquête anonyme à partir d'un cas clinique, simple et rapide, sur vos pratiques cliniques et les mesures que vous prenez chez vos patients avant prescription d'un traitement anti-TNF. Il s'agit de faire un instantané, un état des lieux des pratiques réelles dans notre pays.

Age : |__|__| ans

Sexe F ☐ M ☐

Année d'obtention de la spécialité : |__|__|__|__|

Membre de la SFR : Oui ☐ Non ☐

Modalités actuelles d'exercice :

- libéral exclusif ☐
- praticien attaché hospitalier et exercice libéral ☐
- hospitalier temps plein ☐ temps partiel ☐
- hospitalo-universitaire (PU-PH, PHU, MCU-PH) ☐ CCA ☐ interne de spécialité ☐

Avez-vous déjà prescrit vous-même un traitement anti-TNF ?

Oui ☐ Non ☐

Dans le service hospitalier où vous exercez en consultation ou en salle, disposez-vous d'une liste systématique d'items et d'examens à vérifier avant prescription d'un anti-TNF ?

Oui ☐ Non ☐ Ne sait pas ☐

Numéro d'anonymat : B

Femme, 50 ans, polyarthrite rhumatoïde séropositive (FR IgM) depuis 7 ans avec :

- Echappement à une bithérapie MTX (20 mg/sem PO) + Salazopyrine (2 g/j) + corticothérapie de 10 mg/j + AINS conventionnel à pleine dose.
- Antécédents :
  - ménopause depuis 1 an non substituée, fibro-adénome mammaire
  - hypothyroïdie secondaire à une maladie de Hashimoto
  - Fille de 29 ans atteinte de la même dysthyroïdie
  - Fracture de côte après chute au décours d'un vertige de Ménière
- Examen clinique
  - Pression artérielle = 120/76, auscultation cardiopulmonaire normale
  - Absence de nodule rhumatoïde ou de signe de vascularite

Vous voulez traiter cette patiente par un agent anti-TNF. Quel est votre bilan pré-thérapeutique (en dehors du bilan d'activité ou de sévérité de la maladie) ?

1. Quels antécédents recherchez-vous spécifiquement ?

a. primo-infection ou de tuberculose viscérale	☐
b. fractures	☐
c. antécédent neurologique	☐
d. infections ORL	☐
e. infections cutanées	☐
f. néoplasie ou hémopathie	☐
g. synoviorthèses isotopiques et cortisoniques	☐
h. thrombose veineuse et/ou d'embolie pulmonaire	☐
i. antécédent cardiovasculaire familial	☐
j. cirrhose	☐
k. arthrite septique	☐
l. septicémie	☐
m. bronchopathie chronique	☐
n. antécédent diabétique familial	☐
o. fausses-couches spontanées	☐
p. antécédent néoplasique familial	☐
q. infections broncho-pulmonaires	☐
r. infections urinaires	☐
s. migraine	☐
t. notion d'une positivité d'une IDR à la tuberculine	☐
u. existence de prothèse dentaire	☐
v. qualité d'un traitement anti-tuberculeux antérieur	☐
w. recherche d'un syndrome de Raynaud	☐
x. recherche d'une photosensibilité	☐
y. traitement coronarien et anti-hypertenseur	☐
z. consommation alcoolo-tabagique	☐
aa. notion d'une vaccination par le BCG	☐
bb. existence de prothèses articulaires	☐

Club Rhumatismes & Inflammations

Numéro d'anonymat : B

2. Quelles données de l'examen physique recueillez-vous ?

a. examen ORL	☐
b. recherche d'un livedo	☐
c. auscultation cardiaque	☐
d. examen de l'état veineux	☐
e. auscultation pulmonaire	☐
f. examen des nerfs périphériques	☐
g. examen des paires crâniennes	☐
h. examen des aires ganglionnaires	☐
i. recherche d'une vascularite cutanée	☐
j. examen neurologique des voies longues	☐
k. état buccodentaire	☐
l. examen des téguments	☐

3. Quels examens biologiques, radiologiques et autres prescrivez-vous ?

a. clichés de sinus (4 incidences)	☐
b. électrophorèse des protides sériques	☐
c. anticorps anti-CCP	☐
d. radiographie pulmonaire	☐
e. détection des anticorps antinucléaires	☐
f. recherche d'anticoagulant circulant	☐
g. dosage des transaminases	☐
h. sérologie du CMV	☐
i. phénotypage des sous-populations lymphocytaires	☐
j. sérologie du VIH	☐
k. prescription d'un ECBU	☐
l. recherche des anticorps anticardiolipine	☐
m. sérologie du virus d'Epstein-Barr	☐
n. sérologie d'hépatite C	☐
o. recherche des anticorps anti-ADN	☐
p. recherche d'anticorps antihistones	☐
q. mesure du TP et du TCA/TCK	☐
r. dosage du complément total, des fractions C3 et C4	☐
s. panoramique dentaire	☐
t. échocardiographie ?	☐
u. hémogramme	☐
v. glycémie	☐
w. prescription d'un scanner des sinus	☐
x. ECG	☐

4. Quelles autres mesures préventives prenez-vous ?

a. examen ORL spécialisé	☐
b. consultation pneumologique	☐
c. examen stomatologique spécialisé	☐
d. consultation neurologique	☐
e. consultation cardiologique	☐
f. vaccination anti-grippale	☐
g. IDR à la tuberculine	☐
h. vaccination par le DTTAB	☐
i. vaccination contre le pneumocoque	☐
