# Supplementary material for: Effect of response format for clinical vignettes on reporting quality of physician practice
Source: BMC Health Serv Res. 2009 Jul 28;9:128. doi: 10.1186/1472-6963-9-128 (PMC3224732; doi:10.1186/1472-6963-9-128)
Supplement: Additional file 3 — Questionnaire C. Vignette reporting history of a fictitious 50-year-old woman with active rheumatoid arthritis, candidate for therapy with tumor necrosis factor (TNF) blocking agents, with a closed-ended (multiple-choice) questionnaire about pre-treatment assessment containing only correct items. [file 1472-6963-9-128-S3.pdf]

Club Rhumatismes & Inflammations

Numéro d'anonymat : C

Enquête du CRI

Traitements par agents anti-TNF : quel bilan pré-thérapeutique ?

Frédéric Lioté, Thao Pham, Philippe Ravaud

Les traitements anti-TNF sont prescrits chez des patients atteints de PR ou de spondylarthopathies. Les indications sont actuellement libellées selon les AMM. Ils exposent à un certain nombre d'effets secondaires de sévérité variable pour lesquels il n'y a actuellement en France que peu de recommandations de pratiques ou de prescription d'examens pré-thérapeutiques. Chaque clinicien évalue le cas particulier de son patient selon sa propre expérience.

Au nom du CRI, nous vous proposons de répondre à une enquête anonyme à partir d'un cas clinique, simple et rapide, sur vos pratiques cliniques et les mesures que vous prenez chez vos patients avant prescription d'un traitement anti-TNF. Il s'agit de faire un instantané, un état des lieux des pratiques réelles dans notre pays.

Age : |__|__| ans

Sexe F ☐ M ☐

Année d'obtention de la spécialité : |__|__|__|__|

Membre de la SFR : Oui ☐ Non ☐

Modalités actuelles d'exercice :

- libéral exclusif ☐
- praticien attaché hospitalier et exercice libéral ☐
- hospitalier temps plein ☐ temps partiel ☐
- hospitalo-universitaire (PU-PH, PHU, MCU-PH) ☐ CCA ☐ interne de spécialité ☐

Avez-vous déjà prescrit vous-même un traitement anti-TNF ?

Oui ☐ Non ☐

Dans le service hospitalier où vous exercez en consultation ou en salle, disposez-vous d'une liste systématique d'items et d'examens à vérifier avant prescription d'un anti-TNF ?

Oui ☐ Non ☐ Ne sait pas ☐

Numéro d'anonymat : C

Femme, 50 ans, polyarthrite rhumatoïde séropositive (FR IgM) depuis 7 ans avec :

- Echappement à une bithérapie MTX (20 mg/sem PO) + Salazopyrine (2 g/j) + corticothérapie de 10 mg/j + AINS conventionnel à pleine dose.
- Antécédents :
  - ménopause depuis 1 an non substituée, fibro-adénome mammaire
  - hypothyroïdie secondaire à une maladie de Hashimoto
  - Fille de 29 ans atteinte de la même dysthyroïdie
  - Fracture de côte après chute au décours d'un vertige de Ménière
- Examen clinique
  - Pression artérielle = 120/76, auscultation cardiopulmonaire normale
  - Absence de nodule rhumatoïde ou de signe de vascularite

Vous voulez traiter cette patiente par un agent anti-TNF. Quel est votre bilan pré-thérapeutique (en dehors du bilan d'activité ou de sévérité de la maladie) ?

1. Quels antécédents recherchez-vous spécifiquement ?

a. primo-infection ou de tuberculose viscérale	☐
b. antécédent neurologique familial	☐
c. infections ORL	☐
d. infections cutanées	☐
e. néoplasie ou d'hémopathie	☐
f. antécédent neurologique	☐
g. thrombose veineuse et/ou d'embolie pulmonaire	☐
h. antécédent cardiovasculaire familial	☐
i. cirrhose	☐
j. arthrite septique	☐
k. septicémie	☐
l. antécédent diabétique familial	☐
m. infections broncho-pulmonaires	☐
n. infections urinaires	☐
o. notion d'une positivité d'une IDR à la tuberculine	☐
p. qualité d'un éventuel traitement anti-tuberculeux	☐
q. consommation alcoolo-tabagique	☐
r. notion d'une vaccination par le BCG	☐
s. existence de prothèses articulaires	☐
t. gammapathie monoclonale	☐

2. Quel examen clinique pratiqueriez-vous personnellement ?

a. examen ORL	☐
b. auscultation cardiaque	☐
c. auscultation pulmonaire	☐
d. examen des aires ganglionnaires	☐
e. examen neurologique	☐
f. état buccodentaire	☐
g. examen des téguments	☐

3. Quels examens prescririez-vous ?

a. électrophorèse des protides sériques	☐
b. radiographie pulmonaire	☐
c. détection des anticorps antinucléaires	☐
d. dosage des transaminases	☐
e. sérologie du VIH	☐
f. sérologie d'hépatite C	☐
g. hémogramme	☐

4. Quelles autres mesures prendriez-vous ?

a. IDR 10 Unités	☐
